# Perioperative decline in quantitative and qualitative tear film parameters in clinically healthy mesocephalic *Canis familiaris* under general anesthesia: A prospective study

**DOI:** 10.14202/vetworld.2025.4082-4092

**Published:** 2025-12-27

**Authors:** Liga Kovalcuka, Grēta Elīza Gaile, Laura Voiko, Ilze Dūzena, Madara Nikolajenko, Ivars Lūsis

**Affiliations:** 1 Latvia University of Life Sciences and Technologies, Clinical Institute, Faculty of Veterinary Medicine, Jelgava, LV – 3004 Latvia; 2 Latvia University of Life Sciences and Technologies, Institute of Food and Environmental Hygiene, Faculty of Veterinary Medicine, Jelgava, LV – 3004 Latvia

**Keywords:** canine anesthesia, corneal protection, dog tear film, ophthalmic complications, Schirmer tear test, tear ferning, tear osmolarity, tear film quality

## Abstract

**Background and Aim:**

General anesthesia (GA) suppresses the blink reflex and lacrimal gland activity, making animals more vulnerable to precorneal tear film (PTF) issues. Although decreases in tear volume during GA are well documented, changes in PTF quality are not well understood. This study examined both the quantity and quality of PTF, including the Schirmer Tear Test-1 (STT-1), tear osmolarity (TO), tear ferning (TF), and punctate fluorescein staining (PFS), in healthy mesocephalic *Canis familiaris* undergoing routine non-ophthalmic surgery under GA.

**Materials and Methods:**

A prospective, randomized, pre–post study was conducted on 16 client-owned mesocephalic dogs (32 eyes). All subjects were clinically and ophthalmologically normal and classified as American Society of Anesthesiologists (ASA) I–II. Tear film parameters were evaluated at five perioperative time points: 30 min preoperatively (T0), 10 min post-premedication (T10), 5 min post-induction (T5), at first surgical incision (TS), and at discharge (TD). STT-1, TF, and TO were measured at each time point; PFS was performed at TD. GA consisted of methadone and dexmedetomidine premedication, propofol induction, and isoflurane maintenance. Mixed-effects regression, paired t-tests, and correlation analyses were applied, with p < 0.05 considered significant.

**Results:**

STT-1 values significantly decreased from baseline (21.2 ± 3.3 mm/min) to T10 (13.5 ± 5.9 mm/min; p < 0.001), T5 (6.4 ± 6.3 mm/min; p < 0.001), and TS (0.8 ± 1.6 mm/min; p < 0.001). TO decreased from 374.4 ± 29.3 mOsm/L at T0 to 354.7 ± 28.2 mOsm/L at TS (p < 0.001). TF grades increased from 0.8 ± 1.0 at T0 to 1.5 ± 1.3 at T10 and 2.3 ± 1.4 at T5 (p < 0.001), indicating deterioration of PTF structure. Moderate correlations were observed among STT-1, TF, and TO. At TD, tear parameters remained significantly altered compared with T0, and PFS identified punctate epithelial lesions in 34.4% of dogs. Age showed a moderate negative relationship with STT-1 (b = –0.41 mm/min; p = 0.038).

**Conclusion:**

GA causes a significant decline in the quantity and quality of the PTF, with incomplete recovery by discharge despite the return of spontaneous blinking. These findings emphasize the need for proactive perioperative ocular surface protection and highlight TF and TO as useful early indicators of anesthesia-related ocular surface impairment in mesocephalic *Canis familiaris*.

## INTRODUCTION

Loss of the blink reflex and tear secretion during general anesthesia (GA) increases the risk of precorneal tear film (PTF) deficiencies in animals. The PTF is a complex, dynamic trilaminar structure vital to ocular health. It moisturizes the cornea, clears cellular debris, shields the ocular surface from environmental insults, and supplies nutrients to the avascular cornea [1, 2]. When PTF integrity is compromised, animals risk developing painful ocular surface disorders that can lead to vision loss if not treated [3–5]. Numerous studies have shown a decline in tear production and the development of corneal lesions in dogs during GA [4, 6–8], which can result in longer treatment durations, higher costs, and the need for antimicrobial therapy.

Despite advancements in anesthetic agents and monitoring protocols, perioperative ocular complications remain a significant concern in veterinary practice. Prophylactic topical lubrication greatly decreases these complications [9, 10]; however, data predicting risk factors and qualitative tear film changes remain limited.

PTF compromise during GA is mainly affected by anatomical and physiological factors, especially the inability to fully close the eyelids and the tear-suppressing effects of anesthetic drugs. The Schirmer Tear Test (STT) is commonly used to measure aqueous tear production; however, this study aimed to assess qualitative changes in the tear film using tear ferning (TF), tear osmolarity (TO), and punctate fluorescein staining (PFS) [11–14]. TO is a key indicator of tear film stability and impacts fluid balance, viscosity, and overall ocular surface health, all of which are vital for lubrication and protection [11]. The TF test provides a biochemical overview of the tear film, particularly reflecting the relative concentrations of electrolytes such as sodium, potassium, calcium, and magnesium [11, 15, 16]. PFS highlights disruption of epithelial tight junctions, increased corneal permeability, and ocular surface irritation [14, 17].

Although previous studies have noted reductions in aqueous tear production during GA in dogs, the available literature has mainly focused on measuring tear film quantity using the STT. Very few studies have investigated the quality aspects of the PTF, despite increasing awareness that the biochemical and structural integrity of the tear film, such as osmolarity, crystallization patterns, and epithelial surface condition, also play a vital role in preventing eye injury during surgery. Furthermore, TO and TF, which are increasingly common in both human and veterinary ophthalmology, remain poorly understood in anesthetized dogs. No studies have thoroughly evaluated these parameters across different perioperative time points, nor have they established reference patterns for clinically healthy mesocephalic dogs, which make up a significant portion of the canine population. Importantly, there is limited information on how TO and TF behave during surgery or whether these indicators return to baseline after anesthesia ends. Additionally, little is known about how perioperative changes in PTF quality relate to postoperative epithelial damage, such as PFS. This knowledge gap limits the ability to develop predictive risk models and evidence-based ocular protection protocols for veterinary patients under GA.

This study aimed to thoroughly evaluate perioperative changes in both the quantitative and qualitative characteristics of the PTF in healthy mesocephalic dogs undergoing GA for non-ophthalmic procedures. Specifically, the study sought to: (1) measure aqueous tear production using the STT-1; (2) assess tear quality through TO; (3) analyze structural crystallization patterns with the TF test; and (4) evaluate postoperative corneal epithelial integrity using PFS. All parameters were monitored at several key perioperative time points to track the dynamic changes and partial recovery of the tear film. An additional goal was to investigate the relationships between tear film parameters and demographic factors, such as age, sex, and body weight. By combining these quantitative and qualitative assessments, the study aimed to establish new baseline data, identify early signs of ocular surface issues, and support improved perioperative eye protection strategies in mesocephalic dogs.

## MATERIALS AND METHODS

### Ethical approval

This study was reviewed and approved by the Animal Welfare and Protection Ethics Council at Latvia University of Life Sciences and Technologies (Approval No. LLU_Dzaep_28.08.2024.-1; issued on August 28, 2024). All procedures involving animals were carried out in strict accordance with the national legislation of the Republic of Latvia that governs the protection and humane treatment of animals used for scientific and educational purposes. The study fully adhered to the Animal Research: Reporting of *In Vivo* Experiments 2.0 guidelines to ensure ethical standards, methodological transparency, and reproducibility.

All participating dogs were client-owned animals brought to the University Veterinary Clinic for routine ovariohysterectomy or orchiectomy. Only animals classified as American Society of Anesthesiologists (ASA) physical status I–II were included to reduce health-related risks. Before inclusion, each dog underwent a full clinical and ophthalmic examination to confirm suitability for participation.

Written informed consent was obtained from all pet owners after providing a clear explanation of the study’s purpose, procedures, potential risks, and expected benefits. Owners were assured that participation was completely voluntary and that refusing would not affect the veterinary care their animals received. The study involved no experimental or invasive procedures beyond standard clinical practice, and no additional pain, distress, or discomfort was inflicted on the animals.

During the perioperative assessments, animal welfare was consistently prioritized. Analgesia, anesthesia, and monitoring procedures followed institutional best-practice guidelines to ensure maximum comfort and physiological stability. All animals were monitored throughout the perioperative and recovery periods by licensed veterinarians trained in anesthesia and ophthalmology. No dog was subjected to prolonged restraint, and no animal required euthanasia or emergency intervention due to study procedures.

All animals recovered smoothly and were sent home with standard postoperative instructions. No adverse events or unexpected complications beyond those typical of routine surgical procedures were observed.

### Study period and location

This study was conducted from September 2024 to March 2025 at the Latvia University of Life Sciences and Technologies.

### Study design and animals

This was a prospective and randomized clinical study. Sixteen client-owned mesocephalic dogs (32 eyes) of various breeds were enrolled and randomly assigned to the surgical sequence using a random number generator (Table 1). The study included 6 males and 10 females aged 1 to 8 years, with an average body weight of 19.9 ± 10.7 kg. All dogs were clinically and ophthalmologically healthy.

**Table 1:** Distribution of dog breeds included in the study.

Breed	Number
English Coker Spaniel, 1; Labrador Retriever, 1; Yorkshire Terrier, 1; Doberman, 1; Poodle, 1; Australian Kelpie, 1; German Shepherd, 1; Swiss white dog, 1; German spitz, 1; Russian-European Laika, 1	10
Mixed-breed	6

Inclusion criteria included dogs aged 1–10 years and classified as ASA Class I or II based on the American Society of Anesthesiologists physical status classification [18]. Dogs with ophthalmologic, neurologic, or systemic diseases, those on concurrent medications, or those exhibiting aggressive or uncooperative behavior were excluded. All animals underwent routine ovariohysterectomy or orchiectomy.

### Baseline ophthalmic examinations

Before enrolment, each dog underwent a comprehensive ophthalmic evaluation that included slit-lamp biomicroscopy (Kowa SL19, Japan), direct ophthalmoscopy (Keeler Practitioner, UK), monocular ophthalmoscopy with a PanOptic ophthalmoscope (Welch Allyn, UK), and rebound tonometry (TonoVetPlus®, Finland). All dogs were examined and anesthetized following standardized protocols to ensure consistency across procedures. Ophthalmic tests included STT-1, TF, TO, and PFS.

### Anesthetic and surgical procedures

All dogs were fasted for 6–8 hours before surgery, with free access to water. Upon arrival, behavior was assessed and scored using a previously validated scale [19]. To minimize variability, one anesthetist (LV), one surgeon (LK), and one data collector (GEG) performed all procedures. Surgeries were scheduled between 09:00 and 13:00 to reduce diurnal variation.

Premedication included methadone (0.2 mg/kg IM; Insistor, Austria) and dexmedetomidine (3 μg/kg IM; Dexdomitor, Finland). Meloxicam (0.2 mg/kg SC; Melovem, Netherlands) was administered as a pre-anesthetic. After 10 minutes, the dogs were preoxygenated for 2 min.

Anesthesia was induced using propofol (2–4 mg/kg IV; Anesia, Netherlands), starting at 1 mg/kg, followed by 0.2 mg/kg midazolam, with additional propofol titrated until the loss of palpebral and swallowing reflexes. Maintenance was achieved with isoflurane (Isoflutek, Spain) in 100% oxygen via orotracheal intubation. The EtISO concentration ranged from 1% to 1.5%, targeting 1.1%. Fentanyl (1 μg/kg IV; Latvia) was administered as rescue analgesia when needed.

Physiological parameters, including electrocardiogram, respiratory rate, pulse oximetry (SpO_2_), body temperature, and noninvasive arterial pressure, were monitored continuously. Ventilation was spontaneous unless apnea occurred. Lactated Ringer’s solution was infused at 5 mL/kg/h. End-tidal CO_2_ and EtIso were monitored using a Datex-Ohmeda S/5 multiparameter system. All dogs were positioned dorsally.

### Perioperative tear film assessments

Tear film parameters were recorded at five time points:

- T0: 30 min before surgery (baseline)
- T10: 10 min after premedication
- T5: 5 min after induction
- TS: At first surgical incision
- TD: At discharge, 40 min after surgery

At each time point, STT-1 was performed first, followed by measurements of TF and TO. After full recovery, PFS was conducted to assess corneal epithelial integrity. Demographic variables such as age, sex, body weight, type of surgery, and duration of surgery were recorded.

### STT-1

Tear production was measured using a dye-free STT strip placed in the lateral lower conjunctival fornix of each eye for 1 min, with results recorded in mm/min. Strips were kept behind the eyelids until reaching 20 mm to ensure sufficient tear volume for TF analysis.

### TF test

Ambient temperature and humidity were monitored using a digital thermo-hygrometer. STT-1 strips were processed with modified double-system Eppendorf tubes and centrifuged at 1,843 × *g* for 10 min at 4°C [16]. Two microliters of tear fluid were placed on a glass slide and air-dried for 10 min. Samples were examined at 10× magnification using a Nikon Eclipse Ci-L microscope (Japan), and images were evaluated by three blinded observers (LK, ID, and MN) using Rolando’s grading system [20] and Masmali’s criteria [15].

### Measurement of TO

TO was measured using the TearLab Osmolarity System. Fifty nanoliters of tear fluid were collected from the lower conjunctival sac. Daily calibration was performed with a system test card and monodose saline (300 mOsm/L) prior to measurements.

### PFS

One drop of 1% fluorescein was instilled into each eye, followed by rinsing with 0.9% NaCl. Corneas were examined at 10× magnification under cobalt blue light using a slit-lamp (Kowa SL19). PFS was scored with a modified SPOTS system: 0 (no uptake) to 4 (75%–100% uptake) [21]. During GA, eyelids were taped shut with hypoallergenic tape, and lubrication was deliberately withheld to assess natural PTF decline.

### Statistical analysis

Statistical analyses were performed using StataNow/BE 18.5 (StataCorp, USA). Baseline values were tested for normality with the Shapiro–Francia test and a Box-Cox transformation. Non-normal TO values were further analyzed using the Wilcoxon rank-sum test. Variations in STT, TO, and TF over time points were evaluated with unpaired t-tests with Duncan’s correction; baseline inter-eye differences were compared using paired t-tests. Pearson’s correlation coefficient was used to assess relationships among STT, TF, and TO.

Random-effects generalized least squares regression, with eye as a random intercept, was used to assess the impact of time and demographic variables on tear film parameters. Missing values were not imputed. The sample size (n = 16) was justified through post hoc power analysis using G*Power 3.1.9.6, targeting medium effect sizes with α = 0.05 and power = 0.90. Significance was set at p < 0.05. Data are expressed as mean ± standard deviation (SD).

## RESULTS

### Environmental conditions

Throughout the study period, the examination room temperature ranged from 21.2°C to 24.4°C, with a mean of 23.1°C ± 0.9°C. Relative humidity ranged from 32% to 54%, averaging 43.6 ± 7.7%. These controlled environmental parameters ensured consistency during all perioperative tear film assessments.

### Study population and surgical characteristics

Sixteen client-owned, clinically normal mesocephalic dogs (32 eyes) were included in the study. The cohort consisted of 10 females and 6 males, with a mean age of 3.3 ± 2.7 years and a mean body weight of 19.9 ± 10.7 kg (range: 5.4–39.4 kg). All animals underwent routine ovariohysterectomy or orchiectomy. The average surgical time was 38.1 ± 13.6 min (range: 20–60 min).

### STT findings

**Figure 1:**
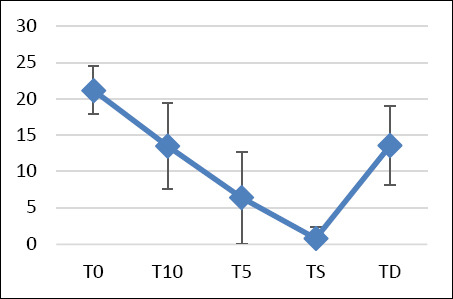
Schirmer tear test during the perioperative period in dogs. T0: baseline value – 30 min before surgery; T10: 10 min after premedication; T5: 5 min after anesthesia induction; TD: discharge time following recovery; TS: during surgery at the time of first cut.

Baseline STT values showed no significant difference between the right and left eyes (*p* = 0.831), with measurements of 21.2 ± 3.5 mm/min and 21.1 ± 3.1 mm/min, respectively (combined: 21.2 ± 3.3 mm/min). Significant decreases in tear production occurred at T10, T5, and TS, with the lowest STT values recorded at TS (right eye: 0.6 ± 1.3 mm/min; left eye: 0.9 ± 1.9 mm/min; combined: 0.8 ± 1.6 mm/min) (Figure 1). At TD, STT values increased but remained significantly lower than baseline (13.6 ± 5.4 mm/min; p < 0.0001) (Table 2).

**Table 2:** Changes in Schirmer tear test (STT), tear osmolarity (TO), and tear ferning grades (TF) throughout the perioperative period in dogs.

Group	STT mm/min ± Standard deviation			Osmolarity ± Standard deviation			Tear ferning ± Standard deviation		
n= 32	OD	OS	OU	OD	OS	OU	OD	OS	OU
T0	21.2	21.1	21.2	365.1	383.8	374.4	0.7	0.8	0.8
	± 3.5	± 3.1	± 3.3	± 38.0	± 11.8	± 29.3	± 0.9	± 1.0	± 1.0
T10	12.1	14.9	13.5	365.6	371.8	368.7	1.6	1.4	1.5
	± 5.7	± 6.0	± 5.9	± 13.4	± 14.0	± 13.8	± 1.5	± 1.1	± 1.3
T5	5.0	7.9	6.4	353.1	356.7	354.9	2.8	1.9	2.3
	± 4.2	± 7.7	± 6.3	± 29.4	± 27.9	± 28.2	± 1.5	± 1.1	± 1.4
TS	0.6	0.9	0.8	334.3	339.9	337.6	-	-	-
	± 1.3	± 1.9	± 1.6	± 32.0	± 26.6	± 28.1	-	-	-
TD	13.0	14.2	13.6	364.9	362.6	363.7	1.6	1.5	1.6
	± 5.0	± 5.8	± 5.4	± 28.2	± 32.3	± 29.9	± 1.3	±± 1.4	± 1.3

Age significantly affected STT outcomes. A moderate negative correlation between baseline STT and age was found (r = –0.476; p = 0.0059). Regression analysis also showed that each additional year of age was linked to a 0.41 mm/min decrease in STT (b = –0.41 mm/min; p = 0.038). Sex, body weight, surgery type, and surgical duration were not related to STT.

### TO findings

**Figure 2:**
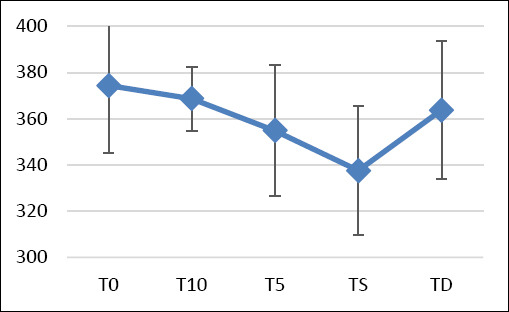
Tear osmolarity during the perioperative period in dogs. T0: baseline value – 30 min before surgery; T10: 10 min after premedication; T5: 5 min after anesthesia induction; TD: discharge time following recovery; TS: during surgery at the time of first cut.

Initial TO values were 365.1 ± 38.0 mOsm/L in the right eye and slightly higher in the left eye (383.8 ± 11.8 mOsm/L), resulting in a combined value of 374.4 ± 29.3 mOsm/L. The lower right eye value was due to a single unexplained outlier (265 mOsm/L; p = 0.030). Significant decreases in TO were observed at T5 and TS, with the lowest values at TS (337.6 ± 28.1 mOsm/L). TO measurements could not be obtained in 14 eyes intraoperatively due to insufficient tear volume (Figure 2). At TD, TO values partially recovered (right: 364.9 ± 28.2 mOsm/L; left: 362.6 ± 32.3 mOsm/L) but remained lower than T0. Changes from baseline were statistically significant only for the left eye (p = 0.0158, parametric; p = 0.0127, nonparametric). Regression analysis showed that time point was the only significant predictor of TO; age, sex, body weight, type of surgery, and surgical duration had no measurable effect.

### TF findings

**Figure 3:**
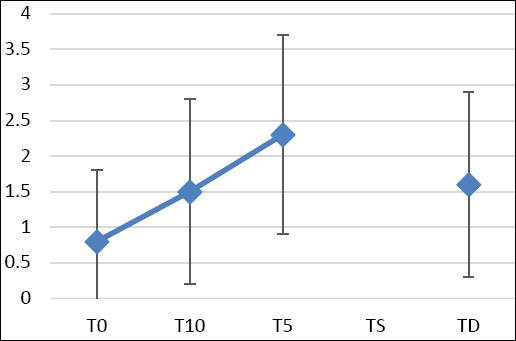
Tear ferning during the perioperative period in dogs. T0: baseline value – 30 min before surgery; T10: 10 min after premedication; T5: 5 min after anesthesia induction; TD: discharge time following recovery; TS: during surgery at the time of first cut.

**Figure 4:**
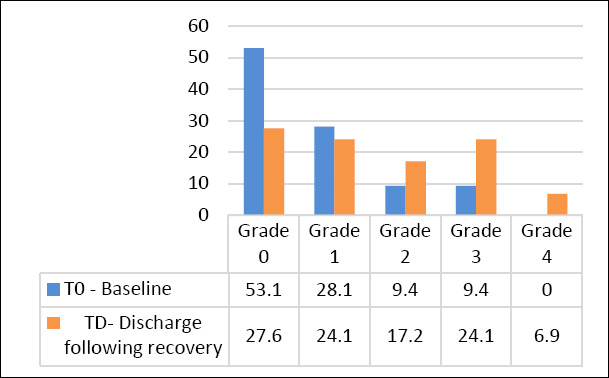
Comparison of tear ferning grades measured before surgery (T0) and at discharge (TD) in dogs (number of eyes, n = 32). A significant difference in TF was found between T0 and TD (p = 0.013).

At baseline, TF grades did not differ between eyes (right: 0.7 ± 0.9; left: 0.8 ± 1.0; combined: 0.8 ± 1.0). TF grades increased significantly at T10 and T5 (Figure 3), indicating deterioration in tear film quality. TF analysis could not be performed at TS because the tear volume was too low. At TD, TF values remained significantly higher than baseline (combined: 1.6 ± 1.3; p = 0.0130) (Table 2). Grade distribution changed noticeably over the course of the study—Grade 0 decreased from 53.1% at T0 to 27.6% at TD, while Grade 4 rose from 0% to 6.9% (Figure 4). Measurement time was the only significant factor influencing TF results. No links were found with age, sex, body weight, surgery type, or surgical duration. Because of missing data at TS, the regression model included 20 eyes over four time points.

### Correlation among tear film parameters

A moderate positive correlation was observed between STT and TO (r = 0.37; p < 0.001) (Table 3). In contrast, TF was moderately negatively correlated with both STT (r = –0.32; p = 0.0006) and TO (r = –0.30; p = 0.0018). These results suggest that lower tear volume and lower osmolarity are associated with poorer TF patterns.

**Table 3:** Correlation of Schirmer Tear Test (STT), tear osmolarity (TO), and tear ferning (TF) values across all perioperative time points.

Precorneal tear film parameters	Pearson’s correlation coefficient	p-value
STT–TO	0.37	0.0000
STT–TF	–0.32	0.0006
TO–TF	–0.30	0.0018

### PFS

**Figure 5:**
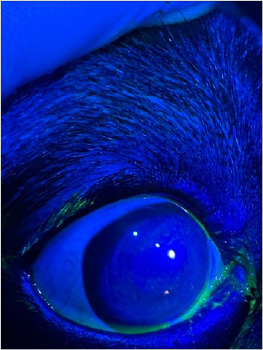
Fluorescein punctate staining at the discharge, indicating stage 1.

At TD, PFS was positive in 34.4% of eyes, mainly with Grade 1 or Grade 2 staining (Figure 5), most often affecting the central corneal area. No corneal ulcers were observed. No corneal ulcers were detected and the relatively mild lesions could be recovered post-discharge

## DISCUSSION

### Overview of tear film alterations during anesthesia

Unlike earlier reports that mainly focused on reductions in aqueous tear volume, the present study offers new insights into the biochemical and structural deterioration of the PTF during and after GA. This broader understanding contributes to a more complete view of perioperative ocular surface physiology. In line with standard clinical protocols, no ocular lubricants were used during GA [22, 23]. However, to prevent additional confounding factors and to ensure precise measurement of natural tear film decline, eyelids were gently closed with hypoallergenic tape throughout anesthesia [24]. To date, data on the incidence of corneal lesions in dogs undergoing GA without eye protection remain limited. In human studies, Grover *et al*. [25] reported that 90% of untreated eyes developed post-anesthetic corneal epithelial defects, compared with only 3.3% of eyes treated with perioperative lubricating ointment.

### Changes in aqueous tear production (STT)

Consistent with previous findings, STT values decreased significantly within 10 minutes after premedication with methadone and dexmedetomidine [26, 27], reaching their lowest levels during surgery. Pietro *et al*. [27] demonstrated that STT values returned to baseline approximately 8 hours after dexmedetomidine-induced sedation. Although STT values increased during recovery in our study, they remained significantly lower than baseline (T0). Prolonged anesthesia is thought to increase the risk of corneal epithelial injury due to prolonged eyelid opening and inadequate tear film replenishment, which can lead to epithelial breakdown and ulceration [28, 29]. Although the procedures in this study averaged 38.1 ± 13.6 min, orthopedic and neurological surgeries may last 2–3 h, posing a higher risk. Previous researchers have also indicated that extended anesthesia or the use of additional drugs in multimodal protocols can affect post-anesthetic tear production and serve as confounding factors [8].

### Qualitative tear film changes assessed by TF

Because the primary goal of this study was to assess qualitative changes in the PTF, TF patterns were examined perioperatively. TF is a sensitive marker of subtle changes in tear biochemistry that are not detectable through clinical examination [15, 30]. According to Rolando *et al*. [20], TF in normal dogs usually appears as types I–II with Masmali Grade 1, while dogs with keratoconjunctivitis sicca show types III–IV [16, 31, 32].

To our knowledge, no previous studies have documented changes in TF during surgery. Our results showed a weak negative correlation between TF and both STT and TO. Initially, most dogs exhibited Grade 0 or 1 TF patterns (53.13% and 28.13%, respectively). After recovery, TF shifted toward higher grades, Grade 1 (27.59%), Grade 2 (17.24%), and mixed Grade 1/3 (24.14%), indicating biochemical instability despite restored blinking and normalized STT values.

An increase in mean TF Grade from 0.8 to 1.6 underscores ongoing tear film deterioration even after recovery. TF does not necessarily indicate disease but signals weakening of PTF quality due to altered biochemical composition.

### TO dynamics and interpretation

TO indicates the concentration of solute osmoles per liter of tear fluid [33, 34] and is strongly linked to inflammatory processes that cause dry eye disease in both humans and dogs [32, 33]. TF patterns are affected by solute concentration, with hyperosmolar tears encouraging dendritic crystal formation [15].

Reported normal TO values in healthy dogs differ depending on the measurement system: 337 ± 16.2 mOsm/L and 340 ± 15.9 mOsm/L with TearLab®, and 293–315 mOsm/L with I-PEN® [12, 13, 35–37]. TearLab® usually shows higher TO values in dogs compared to I-PEN®, which is opposite to the pattern seen in humans [38, 39].

In this study, baseline TO values were comparatively higher (374.4 mOsm/L) and showed a mild decrease during GA followed by partial recovery postoperatively. A weak positive correlation with STT was identified. Although GA inevitably suppresses blinking and lacrimal secretion, limited veterinary research has examined intraoperative TO fluctuations. In human medicine, TO significantly increases one week after cataract surgery and may remain elevated for several weeks [40].

### Incidence of postoperative corneal epithelial compromise

At discharge, 34% of dogs showed positive fluorescein punctate staining (PFS), mainly grades 1 and 2, despite having no obvious clinical signs. Previous studies report PFS in 10% of dogs immediately after extubation and 6%–18.6% at 24 hours postoperatively when different lubricant regimens were compared [22, 23].

A large retrospective study of 732 dogs reported a 1.9% incidence of GA-associated corneal ulceration, with two cases progressing to severe complications [4]. In pediatric human patients, postoperative ocular complications included punctate epithelial erosions (2.7%), combined erosions and reduced tear breakup times (2.7%), and unilateral corneal abrasion (0.9%). Risk factors include young age, prolonged surgery, and procedures involving the head and neck region [41].

### Clinical implications and ocular protection recommendations

Although tear quantity begins to improve after surgery, our findings indicate that tear quality continues to decline postoperatively. Combined assessment of TF and TO may serve as early markers of ocular surface compromise during anesthesia, detecting changes that may occur before measurable decreases in STT.

To reduce the risk of corneal injury, postoperative ocular protection is crucial. While eyelid taping and lubrication help decrease exposure, they do not promote sufficient tear production and may not fully prevent keratopathy. Based on our findings, we recommend:

- Applying ocular lubricants immediately after intubation,
- Maintaining lubrication throughout surgery, and
- Reapplying lubrication during recovery.

Consistent perioperative use of ophthalmic lubricants is highly recommended for all veterinary patients, regardless of the procedure type or whether standardized protocols are available.

## CONCLUSION

This prospective study shows that GA causes significant quantitative and qualitative deterioration of the PTF in clinically healthy mesocephalic dogs. A notable reduction in STT values occurred within 10 minutes after premedication, with intraoperative values approaching near-zero levels (0.8 ± 1.6 mm/min). TO also decreased significantly during surgery and remained below baseline at discharge, while TF grades increased from a preoperative mean of 0.8 to 1.6, indicating persistent biochemical instability despite recovery of the blink reflex. Additionally, 34.4% of dogs showed PFS at discharge, reflecting subtle corneal epithelial damage. Age was the only demographic factor associated with reduced tear production, suggesting increased vulnerability in older dogs.

These findings show that anesthesia-induced PTF disruption is more complex and widespread than previously thought, affecting both tear volume and tear quality. From a clinical standpoint, proactive eye protection during the perioperative period is critical. Applying ophthalmic lubricants early at induction, maintaining lubrication during surgery, and reapplying as needed during recovery should be standard parts of anesthetic protocols to reduce the risk of postoperative keratopathy and protect the ocular surface.

This study offers notable strengths, including the integration of four complementary diagnostic tools, STT, TO, TF, and fluorescein staining, and the evaluation of five perioperative time points, resulting in the first intra-anesthetic reference dataset for tear quality dynamics in mesocephalic dogs. However, limitations include a small sample size, the absence of a non-anesthetized control group, and a lack of extended postoperative follow-up beyond discharge.

Future research should examine tear film recovery at 24 h and one week after surgery, assess the relative risks in brachycephalic breeds and dogs with existing ocular conditions, and evaluate the effectiveness of various lubrication methods and anesthetic protocols in maintaining tear film stability.

Overall, GA causes a significant and clinically meaningful disruption of the canine tear film, with incomplete recovery by discharge. Consistent perioperative ocular lubrication is highly recommended to lower the risk of anesthesia-related corneal injury and to improve ocular safety in veterinary patients.

## DATA AVAILABILITY

The supplementary data can be made available from the corresponding author upon request.

## AUTHORS’ CONTRIBUTIONS

LK, LV, and ID: Contributed to the conception and design of the study. LK, ID, LV, IL, GEG: Data and sample collection, data analysis and interpretation, and manuscript drafting, editing, and revision. MN: Conducted laboratory work, tear ferning test. LK: Supervised the study and edited the manuscript. IL: Statistical data analysis.

## References

[1] Corsi F, Arteaga K, Corsi F, Masi M, Cattaneo A, Selleri P, Crasta M, Peruccio C, Guandalini A (2022). Clinical parameters obtained during tear film examination in domestic rabbits. BMC Vet. Res.

[2] Vitor RC, de Carvalho Teixeira JB, Dos Santos KC, Oliveira GMS, Guedes PEB, da Paixão Sevá A, Gomes Junior DC, Veloso JF, Carlos RSA (2024). Shih-Tzu dogs show alterations in ocular surface homeostasis despite adequate aqueous tear production. Acta Vet. Scand.

[3] Kaye AD, Renschler JS, Cramer KD, Anyama BO, Anyama EC, Gayle JA, Armstead Williams CM, Mosieri CN, Saus JA, Cornett EM (2019). Postoperative management of corneal abrasions and clinical implications:a comprehensive review. Curr. Pain Headache Rep.

[4] Park YW, Son WG, Jeong MB, Seo K, Lee LY, Lee I (2013). Evaluation of risk factors for development of corneal ulcer after nonocular surgery in dogs:14 cases 2009–2011. J. Am. Vet. Med. Assoc.

[5] Yoshikawa Y, Yokoi N, Kato H, Sakai R, Komuro A, Sonomura Y, Ikeda T, Sotozono C (2021). Evaluation of eye-pain severity between dry-eye subtypes. Diagnostics.

[6] Zernii EY, Baksheev VE, Kabanova EI, Tiulina VV, Golovastova MO, Gancharova OS, Savchenko MS, Sotikova LF, Zamyatnin AA, Filippov PP, Senin II (2018). Effect of general anesthesia duration on recovery of secretion and biochemical properties of tear fluid in the post-anesthetic period. Bull. Exp. Biol. Med.

[7] Herring IP, Pickett JP, Champagne ES, Marini M (2000). Evaluation of aqueous tear production in dogs following general anesthesia. J. Am. Anim. Hosp. Assoc.

[8] Shepard MK, Accola PJ, Lopez LA, Shaughnessy MR, Hofmeister EH (2011). Effect of duration and type of anesthetic on tear production in dogs. Am. J. Vet. Res.

[9] Dawson C, Sanchez RF (2016). Prospective study of the prevalence of corneal surface disease in dogs receiving prophylactic topical lubrication under general anesthesia. Vet. Ophthalmol.

[10] Li S, Lei G, Liu Y, Tian L, Jie Y, Wang G (2024). The protective effect of vitamin A palmitate eye gel on the ocular surface during general anaesthesia surgery:a randomized controlled trial. Int. Ophthalmol.

[11] Wolffsohn JS, Benítez-Del-Castillo J, Loya-Garcia D, Inomata T, Iyar G, Liang L, Pult H, Sabater AL, Starr CE, Vehof J, Wang MT, Chen W, Craig JP, Dogru M, Quinones VLP, Stapleton F, Sullivan DA, Jones L (2025). TFOS DEWS III diagnostic methodology. Am. J. Ophthalmol.

[12] Leonard BC, Stewart KA, Shaw GC, Hoehn AL, Stanley AA, Murphy CJ, Thomasy SM (2019). Comprehensive characterization of the ocular surface in spontaneous aqueous deficient dry eye disease in dogs. Cornea.

[13] Sebbag L, Silva APSM, Santos APB, Raposo ACS, Oriá AP (2023). An eye on the Shih-Tzu dog:Ophthalmic examination findings and ocular surface diagnostics. Vet. Ophthalmol.

[14] Iwashita H, Sebbag L, Leonard BC, Saito A (2023). A review of diagnostic tests for qualitative and quantitative tear film deficiency in dogs. Vet. Ophthalmol.

[15] Masmali AM, Purslow C, Murphy PJ (2014). The tear ferning test:a simple clinical technique to evaluate the ocular tear film. Clin. Exp. Optom.

[16] Oriá AP, Raposo ACS, Araújo NLLC, Lima FB, Masmali AM (2018). Tear ferning test in healthy dogs. Vet. Ophthalmol.

[17] Mokhtarzadeh M, Casey R, Glasgow BJ (2011). Fluorescein punctate staining traced to superficial corneal epithelial cells. Invest. Ophthalmol. Vis. Sci.

[18] Hurwitz E, Simon M, Vinta S, Zehm C, Shabot S, Minhajuddin A, Abouleish A (2017). Adding examples to the ASA-physical status classification improves correct assignment. Anesthesiology.

[19] Romano M, Portela D, Breghi G, Otero P (2016). Stress-related biomarkers in dogs administered regional anaesthesia or fentanyl during stifle surgery. Vet. Anaesth. Analg.

[20] Rolando M (1984). Tear mucus ferning test in normal and keratoconjunctivitis sicca eyes. Chibret Int. J. Ophthalmol.

[21] Saito A, Iwashita H, Kitamura Y, Miwa Y, Arita R (2021). Punctate fluorescein staining scores in dogs with or without aqueous tear deficiency. Vet. Ophthalmol.

[22] Di Palma C, Micieli F, Lamagna B, Nieddu A, Uccello V, Fatone G, Vesce G (2020). Schirmer tear test value and corneal lesions during general anesthesia in non-brachycephalic dogs. Vet. Sci.

[23] Giannetto C, Macrì F, Falcone A, Giudice E, Crupi R, Cicero L, Cassata G, Staffieri F, Di Pietro S (2021). Evaluation of tear production as measured by schirmer test i in dogs after acepromazine and acepromazine–methadone premedication. Animals.

[24] Ioannides J, Parker J, Kumaratunga V, Preston J, Donaldson D, MacFarlane P, Hartley C (2022). Comparison of corneal injury incidence using two protection methods in dogs under general anesthesia. Vet. Ophthalmol.

[25] Grover VK, Kumar KV, Sharma S, Sethi N, Grewal SP (1998). Comparison of methods of eye protection under general anaesthesia. Can. J. Anaesth.

[26] Raušer P, Mrázová M, Novák L, Staňková L, Pavlík M (2022). Influence of methadone on ocular parameters in healthy dogs. Top. Companion Anim. Med.

[27] Di Pietro S, Giannetto C, Falcone A, Piccione G, Congiu F, Staffieri F, Giudice E (2021). Dexmedetomidine and tear production in dogs. Vet. Sci.

[28] Sanchez RF, Mellor D, Mould J (2006). Effects of medetomidine and medetomidine–butorphanol combination on Schirmer tear test readings in dogs. Vet. Ophthalmol.

[29] Stanley RG (2009). Corneal ulceration associated with general anesthesia in dogs:Risk factors and outcome. J. Am. Anim. Hosp. Assoc.

[30] Pearce EI, Tomlinson A (2000). Spatial location studies on chemical composition of human tear ferns. Ophthalmic Physiol. Opt.

[31] Williams D, Hewitt H (2017). Tear ferning in normal dogs and dogs with keratoconjunctivitis sicca. Open Vet. J.

[32] Lamkin ID, Zimmerman KL, Smith Fleming KM, Martins BC (2020). Osmolarity of basal and reflex tears of normal dogs. Vet. Ophthalmol.

[33] Lemp MA, Bron AJ, Baudouin C, Benítez-Del-Castillo JM, Geffen D, Tauber J, Foulks GN, Pepose JS, Sullivan BD (2011). Tear osmolarity in the diagnosis and management of dry eye disease. Am. J. Ophthalmol.

[34] Murube J (2006). Tear osmolarity. Ocul. Surf.

[35] Ng ATJ, Moore PA, Boveland SD (2024). Meibography, tear lipid layer and osmolarity in healthy dogs. Vet. Ophthalmol.

[36] Kim HW, Kim JY (2023). Randomized comparison of TearLab®and I-PEN®osmometry in normal dogs. Vet. Ophthalmol.

[37] Sebbag L, Park SA, Kass PH, Maggs DJ, Attar M, Murphy CJ (2017). Tear film osmolarity using the TearLab osmometer in normal and KCS dogs. Vet. Ophthalmol.

[38] Alanazi MA, El-Hiti GA, Alhafy N, Almutleb ES, Fagehi R, Alanazi SA, Masmali AM (2022). Correlation between osmolarity measurements using TearLab and I-Pen in high BMI subjects. Adv. Clin. Exp. Med.

[39] Alanazi MA, El-Hiti GA, Alturki OA, Alanazi M, Fagehi R, Masmali AM (2022). Tear osmolarity in smokers using TearLab and I-Pen systems. J. Ophthalmol, 2022.

[40] Igarashi T, Takahashi H, Kobayashi M, Kunishige T, Arima T, Fujimoto C, Suzuki H, Okuda T, Takahashi H (2021). Changes in tear osmolarity after cataract surgery. J. Nippon Med. Sch.

[41] El Hadi D, Hoyeck S, Rachid E, El Moussawi Z, Torbey J, Aouad M, Al-Haddad C (2024). Ocular surface complications in children undergoing general anaesthesia. J. Perioper. Pract.

